# Whole blood impedance aggregometry as a biomarker for the diagnosis and prognosis of severe sepsis

**DOI:** 10.1186/cc11816

**Published:** 2012-10-22

**Authors:** Michael Adamzik, Klaus Görlinger, Jürgen Peters, Matthias Hartmann

**Affiliations:** 1 Duisburg-Essen, Hufelandstr. 55, 45122 Essen, Germany

## Abstract

**Introduction:**

Sepsis leads to an activation of the immune system and hemostatis. However, studies on platelet aggregation in severe sepsis using impedance aggregometry have not been performed and the diagnostic and prognostic capabilities are unknown. In the present study we hypothesized that impedance aggregometry findings might serve as a biomarker for the diagnosis and prognosis of severe sepsis.

**Methods:**

Eighty patients with severe sepsis and 50 postoperative patients were included in the prospective observational study. Platelet function was determined at the first day of severe sepsis and surgery, respectively, using impedance aggregometry (Multiplate^®^). Moreover, platelet count, procalcitonin, interleukin 6, C-reactive protein and 30-day mortality were determined.

**Results:**

Compared to postoperative patients, platelet aggregation was significantly reduced in patients with severe sepsis (collagen-test: 70.8 (44.4, 83.2) arbitrary units (A.U.) vs. 26.8 (12.7, 45.8) A.U.; *P *<0.001; median and quartiles). Furthermore, marked differences in platelet function were observed in survivors and non-survivors of severe sepsis (collagen-test: 33.4 (10.9, 48.8) A.U. vs. 12.4 (6.5, 25.0) A.U.; *P *= 0.001). Kaplan-Meier analysis demonstrated that higher platelet function was associated with a mortality of 10%, while mortality was 40% when platelet function was low (collagen-test; *P *= 0.002). The odds ratio was 6.0. In both univariate and multivariate analyses (including procalcitonin, IL6, C-reactive protein and platelet count) impedance aggregometry using collagen as the activator proved to be the best and an independent predictor for the diagnosis and prognosis of severe sepsis in critical illness.

**Conclusions:**

In severe sepsis, impedance aggregometry allows better prediction of diagnosis and survival than conventional biomarkers and platelet count.

## Introduction

Sepsis is the third most common cause of death in western countries. Despite the advances in intensive care medicine the prognosis of the disease has improved only gradually [1]. Concerning the pathophysiology, sepsis has been shown to be caused by a generalized and inappropriate activation of the immune system and hemostasis; both plasma components and platelets are affected by the disease [2].

In recent years, evidence has accumulated that platelets and inflammation are tightly coupled. An active function of platelets in inflammation has recently been demonstrated: several studies suggest that platelets serve as circulating sentinels that bind infectious agents and present them to the reticuloendothelial system [3-5]. Interestingly, it has been demonstrated that Toll-like receptors 1, 2, 4 and 9 are localized on the surface of human platelets, and thus an effect of endotoxins on platelets in sepsis has been hypothesized [6]. Indeed, the evidence is growing that activation of TLRs is responsible for the LPS-induced thrombocytopenia and TNF-alpha production [7]. Experimental findings on platelet aggregation due to lipopolysaccharide and bacteria, however, are contradictory. In a rat endotoxin model, a decreased ADP-induced platelet aggregation was determined and bacterial products were shown to inhibit platelet function in human platelets [8-11]. In contrast, a recent study demonstrates in an *ex vivo *setting that bacteria isolated from patients with Gram-positive sepsis can induce platelet aggregation [12]. This notion was confirmed by Rasmussen *et al*. who described that clinical isolates from *Enterococcus faecalis *can aggregate human platelets [13]. However, in another study, differences in the pro-aggregatory effects were observed between bacterial strains and individuals [14].

A new device for the measurement of platelet function using whole blood impedance aggregometry as the principle is available [15]. The Multiplate^® ^device (Verum Diagnostica, Munich, Germany) allows the simultaneous measurement of whole blood samples with five activators and the computerized design makes a point of care measurement possible. The device has been demonstrated to be a valuable tool in different clinical settings. In cardiology, impedance aggregometry is predictive of stent thrombosis and early mortality following percutaneous coronary intervention [16]. In neuroradiology, the device was used to predict stent thrombosis and adverse events [17]. Moreover, impedance aggregometry is predictive of blood loss in cardiac surgery [18]. However, impedance aggregometry findings in patients with sepsis have not been investigated.

It was the aim of the present study to investigate the effects of severe sepsis on platelet function using impedance aggregometry. We hypothesized that impedance aggregometry might serve as a biomarker for the diagnosis and prognosis of severe sepsis in critical illness.

## Materials and methods

### Patients

This study was formally and specifically reviewed and approved by the Ethics Committee of the University Hospital Essen, the appropriate institutional review board. Informed written consent was given by postoperative patients. Informed consent of patients with sepsis was waived by the ethics committee, but written informed consent for the use of data was acquired from the surviving patients after recovery from the disease. Eighty patients admitted to an ICU of the University Hospital Essen were considered eligible for the prospective observational study, if they fulfilled the criteria for severe sepsis as recently defined [19]. As the control group, 50 patients admitted to the ICU after surgery but without meeting criteria of sepsis were chosen. Within 24 hours, blood was drawn and procalcitonin, interleukin 6, C-reactive protein, platelet count, international normalized ratio (INR), thrombin time and fibrinogen were determined. Moreover, simplified acute physiology score (SAPS II) and sequential organ failure assessment score (SOFA) were calculated over the first 24 h after the patient met the severe sepsis criteria [20,21]. All patients were followed-up at 30 days for survival. Details of patients' characteristics are given in Table 1.

**Table 1:** Characterization of postoperative patients and patients with severe sepsis (survivors, non-survivors, all)

Patient characteristics	Postoperative patients mean and SD *median (quartiles)*	Survivors of sepsis mean and SD *median (quartiles)*	Non-survivors of sepsis mean and SD *median (quartiles)*	All patients with sepsis mean and SD *median (quartiles)*
Patient number (n =)	50	60	20	80
Age	57.1 ± 17.2 *65.0 (47.0, 70.0)*	58.8 ± 16.7 *60.0 (46.0, 74.0)*	53.4 ± 14.1 *53.0 (47.5, 64.5)*	57.5 ± 16.1 *57.0 (47.3, 69.0)*
Gender	23/27	40/20	9/11	49/31
**Primary diagnosis**				
GI disease	6	15	7	22
GI cancer	14	7	4	11
Cancer other	6	4	3	7
Urogenital disease	3	2	1	3
Urogenital cancer	7	1	1	2
Cardiovascular	10	18	2	20
Lung disease	0	9	1	10
Lung cancer	0	1	0	1
Other diseases	4	3	1	4
**Infection type**				
Gram-pos	0	21	8	29
Gram-neg	0	21	6	27
Fungal	0	6	2	8
None	0	12	4	16

Included are biometric data, primary diagnosis, and infection type obtained within 24 h of ICU admission based on 30-day mortality. Values are given both as mean and standard deviation as well median and quartiles.

### Impedance aggregometry

For the platelet function tests, heparinized samples were subjected to the Multiplate^® ^analysis according to the manufacturer´s recommendations. A total of 300 μl saline and 300 μl heparinized whole blood were added to the test cell. After three minutes of incubation at 37°C, samples were activated with arachidonic acid, adenosine diphosphate (ADP), collagen or thrombin receptor activating peptide 6 (TRAP) according to the manufacturer´s recommendations (Verum Diagnostica, Munich, Germany). Normal values of aggregometry are given in the appropriate tables. Platelet aggregation findings were assessed by determination of the area under curve in arbitrary units (A.U.).

### Assays for procalcitonin, interleukin 6, and C-reactive protein concentrations

For the determination of procalcitonin concentration, the Liaison Brahms PCT assay (Diasorin S.p.A., Sallugia, Italy) was used. C-reactive protein was measured with the CRP wide-range assay of the Avidia 1650 chemistry system (Bayer Healthcare, LLC, Leverkusen, Germany). Interleukin 6 was determined by using an Immulite 2000 systems analyzer and reagents (Siemens Healthcare Diagnostics Products, Ltd., Duisburg, Germany).

### Statistical assessments

Values are given as mean and standard deviation as well as median and quartiles. For the statistical evaluation of differences between groups, the Mann-Whitney test was used since normal distribution of several values was excluded by the Shapiro-Wilk test. Receiver operating characteristic curves and the correspondent areas under the curve, the asymptotic significances and the 95% asymptotic confidence intervals were used for the univariate comparison of impedance aggregometry findings with conventional biomarkers. Correlation coefficients given in the results are derived from linear correlation analyses. Dependence of 30-day survival on impedance aggregometry findings was evaluated using Kaplan-Meier-analysis. The median of the respective variable was used as the cut-off to discriminate groups with low and high values, respectively. Furthermore, the odds ratios were determined, as appropriate.

The diagnostic and prognostic value of impedance aggregometry, conventional biomarkers and platelet count was also determined by multivariate analyses using logistic regression. SPSS Version 19 (SPSS Inc., Chicago, IL, USA) was used for all statistical procedures. An *a priori *alpha error *P *of less than 0.05 was considered to indicate statistical significance.

## Results

### Impedance aggregometry as a biomarker for the diagnosis of severe sepsis in critical illness

**Figure 1:**
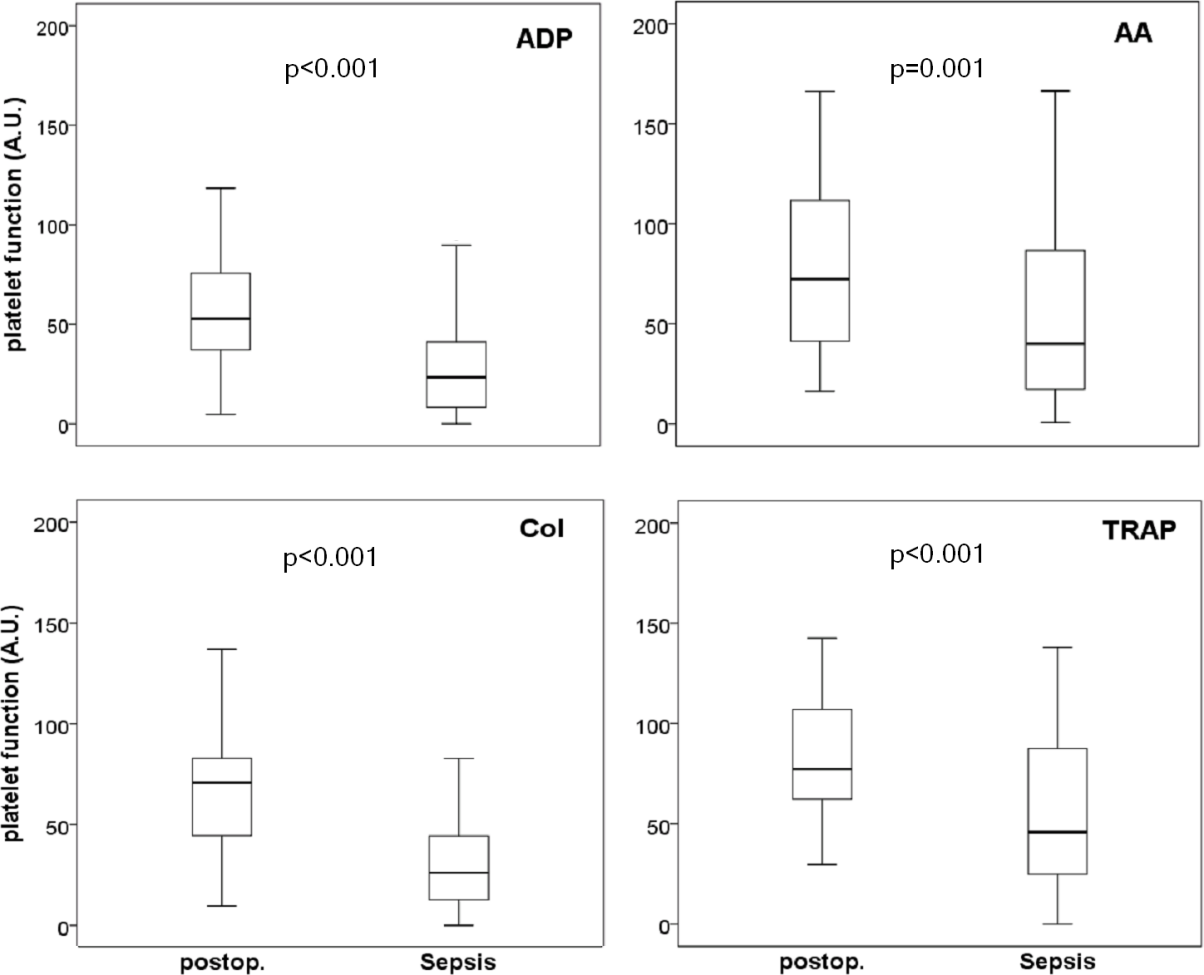
**Platelet aggregation, as determined by impedance aggregometry, in postoperative and septic patients**. Shown are the aggregometry findings (area under curve (AUC) in arbitrary units (A.U.)) with the activators adenosine diphosphate (ADP), collagen (COL), arachidonic acid (AA) and thrombin receptor activating peptide 6 (TRAP). Results are given as boxplots (with median, quartiles, minimum and maximum). For the statistical evaluation the Mann-Whitney test was used.

**Figure 2:**
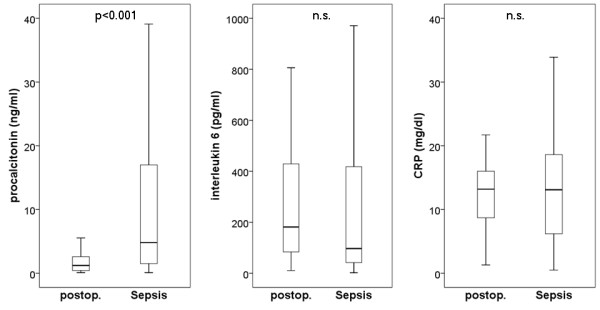
**Conventional sepsis marker in postoperative patients and patients with severe sepsis**. Values for procalcitonin, interleukin 6 (IL-6), and C-reactive protein are given as boxplots (with median, quartiles, minimum and maximum). The Mann-Whitney test was used for statistical evaluation.

Platelet aggregation was determined in 80 patients with severe sepsis and in 50 postoperative patients, respectively. The results demonstrate decreased platelet aggregation in patients with severe sepsis as assessed with the four activators (Figure 1). Moreover, the conventional biomarkers of inflammation, namely procalcitonin, interleukin 6 and C-reactive protein were determined. The results, shown in Figure 2, demonstrate that procalcitonin is capable of detecting patients with severe sepsis in critical illness, while C-reactive protein was elevated in both postoperative patients and patients with severe sepsis. Interleukin 6 was even lower in patients with severe sepsis (when compared with postoperative patients). Data on impedance aggregometry findings, conventional sepsis markers, ICU scores and conventional coagulation variables in postoperative patients and patients with severe sepsis are summarized in Table 2.

**Table 2:** Impedance aggregometry findings, sepsis markers, ICU-scores, and coagulation variables in postoperative and septic patients.

**Test ***normal range*	**Postop. patients **mean and SEM *median (quartiles)*	**Patients with sepsis **mean and SEM *median (quartiles)*	**Significance level ***Mann-Whitney-test*
**Aggregometry**			

**ADP-test ***53 - 122 AU*	55.66 ± 3.80 *52.85 (36.7, 76.00)*	31.87 ± 3.50 *23.70 (8.30, 42.68)*	*<0.001*
**AA-test ***74 - 136 AU*	78.08 ± 5.71 *72.40 (41.78, 111.75)*	54.65 ± 4.81 *41.10 (18.30, 87.72)*	*0.001*
**Col-test ***46 - 116 AU*	68.13 ± 4.18 *70.75 (44.38, 83.22) *	35.28 ± 3.52 *26.75 (12.73, 45.75) *	*<0.001*
**TRAP-test ***94 - 156 AU*	83.52 ± 4.38 *77.30 (60.10, 108.65) *	58.20 ± 4.38 *47.55 (25.08, 89.45) *	*<0.001*

**Sepsis marker**			

**Procalcitonin ***0 - 0.5 ng/ml *	2.09 ± 0.41 *1.2 (0.44, 2.60)*	18.87 ± 5.13 *4.55 (1.45, 17.60)*	*<0.001*
**IL 6 ***0 - 3.4 pg/ml *	297.11 ± 40.95 *181.50 (83.50, 439.50)*	788.97 ± 310.20 *95.85 (40.75, 401.50) *	*0.045*
**CRP ***0 - 0.5 mg/dl *	12.18 ± 0.81 *13.20 (8.55, 16.05) *	13.79 ± 1.02 *13.10 (6.13, 18.58) *	*0.813*

ICU-Scores			

SAPSII	21.4 ± 9.4 *22.0 (15.5, 29.0)*	48.9 ± 17.1 *51.0 (36.0, 65.0)*	*<0.001*
SOFA	3.4 ± 2.5 *4.0 (1.0, 6.0)*	11.6 ± 4.6 *12.0 (8.0, 15.0)*	*<0.001*

**Coagulation variables**			

**INR ***0.89 - 1.11*	1.19 ± 0.02 *1.17 (1.08, 1.29)*	1.38 ± 0.06 *1.27 (1.11, 1.48)*	*0.029*
**Thrombin time ***14 - 21 s*	18.1 ± 0.7 *17.0 (15.8, 19.1)*	23.9 ± 1.7 *18.6 (15.3, 24.8)*	0.105
**Fibrinogen ***210 - 400 mg/dl*	406 ± 27 *364 (312, 495)*	479 ±30 *471 (240, 662)*	*0.285*
**Platelets ***160 - 350/nl*	207 ± 14 *196 (154, 245)*	137 ± 12 *107 (57, 187)*	*<0.001*

For the activation of platelet aggregation adenosine diphosphate (ADP), arachidonic acid (AA), collagen (Col) and thrombin receptor activating peptide (TRAP) were used. Data are given as mean and standard error of the mean as well as *median and quartiles*. For the statistical evaluation the Mann-Whitney test was used.

**Figure 3:**
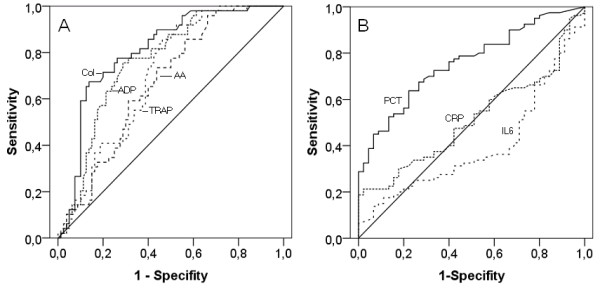
**Impedance aggregometry findings (A) and conventional sepsis markers (B) in postoperative patients and patients with severe sepsis**. Shown are the receiver operating characteristic curves. The activators used for impedance aggregometry were adenosine diphosphate (ADP), collagen (COL), arachidonic acid (AA) and thrombin receptor activating peptide (TRAP). The conventional biomarkers were procalcitonin (PCT), interleukin 6 (IL-6), and C-reactive protein (CRP).

To further compare impedance aggregometry with conventional biomarkers, receiver operating characteristic curves were generated from the variables (Figure 3). The areas under the curve as a measure of assay reliability obtained from these curves as well as the significance levels and the confidence intervals are shown in Table 3. The results demonstrate that impedance aggregometry using collagen as the activator was a better biomarker for the diagnosis of severe sepsis in critical illness than procalcitonin, interleukin 6 and C-reactive protein. The results are confirmed by multivariate analyses. Inclusion of procalcitonin, interleukin 6 and C-reactive protein, as well as impedance aggregometry findings obtained by one of the four activators, demonstrated that impedance aggregometry findings obtained with the activators ADP (*P *= 0.009), collagen (*P *= 0.002) and thrombin receptor activating peptide (*P *<0.001), but not arachidonic acid, were independent predictors of severe sepsis in critical illness. Comparison of the four activators with procalcitonin, interleukin 6, and C-reactive protein in one analysis demonstrated that impedance aggregometry using collagen as the activator (*P *= 0.002) was the best predictor of severe sepsis in critical illness and thus confirm the results of univariate analyses.

**Table 3:** ROC-curve statistics for impedance aggregometry, sepsis markers, ICU-scores and coagulation variables in postoperative and septic patients

Diagnosis of sepsis Test	AUC	Confidence interval	Significance level
**Aggregometry**			

ADP	0.759	0.677 - 0.842	<0.001
AA	0.669	0.577 - 0.761	0.001
Col	0.808	0.731 - 0.884	<0.001
TRAP	0.700	0.661 - 0.778	<0.001

**Sepsis marker**			

PCT	0.754	0.674 - 0.840	<0.001
IL6	0.604	0.296 - 0.497	0.055
CRP	0.513	0.412 - 0.614	0.813

**ICU-scores**			

SAPS	0.915	0.866 - 0.963	<0.001
SOFA	0.926	0.880 to 0.971	0.001

**Coagulation variables**			

INR	0.617	0.518 - 0.715	0.029
Thrombin time	0.593	0.456 - 0.669	0.285
Fibrinogen	0.563	0.456 - 0.667	0.285
Platelets	0.736	0.649 - 0.823	<0.001

Area under curve, asymptotic confidence interval as well as asymptotic *P*-values are given for impedance aggregometry using the activators adenosine diphosphate (ADP), arachidonic acid (AA), collagen (Col) and thrombin receptor activating peptide (TRAP), as well as for the conventional sepsis markers procalcitonin (PCT), interleukin 6 (IL 6) and C-reactive protein (CRP).

### Impedance aggregometry as a biomarker for the prognosis of severe sepsis in critical illness

Comparison of platelet function in survivors and non-survivors of sepsis demonstrated significant lower impedance aggregometry values in non-survivors when ADP, collagen or TRAP were used as the activators. When arachidonic acid was used as the activator, no significant difference was detectable. Furthermore, procalcitonin- and IL-6 concentrations were higher in non-survivors. Details of impedance aggregometry findings, conventional sepsis markers, ICU scores and coagulation variables in survivors and non-survivors of severe sepsis are summarized in Table 4. The correspondent analyses of receiver operating characteristic curves for these variables are shown in Table 5.

**Table 4:** Impedance aggregometry findings, sepsis markers, ICU-scores, and coagulation variables, in survivors and non-survivors of severe sepsis

**Test ***normal range*	**Survivors ***mean and SEM median (quartiles)*	**Non-survivors ***mean and SEM median (quartiles)*	Significance level Mann-Whitney test
**Aggregometry**			

**ADP-test ***53 - 122 AU*	37.35 ± 4.39 *32.30 (9.33, 48.98)*	15.03 ± 3.03 9.65 (4.45, 22.43)	*0.004*
**AA-test ***74 - 136 AU*	59.82 ± 5.75 *43.45 (22.35, 93.85)*	39.15 ± 7.71 *25.50 (10.03, 60.75)*	0.059
**Col-test ***46 - 116 AU*	40.89 ± 4.29 *33.40 (10.90, 48.78)*	18.43 ± 3.79 12.40 (6.50, 24.95)	*0.001*
**TRAP-test ***94 - 156 AU*	66.76 ± 5.09 *54.75 (33.20, 97.08)*	32.50 ± 5.59 24.45 (13.20, 56.33)	*<0.001*

**Sepsis marker**			

**Procalcitonin ***0 - 0.5 ng/ml *	17.74 ± 6.22 *3.39 (1.14, 17.35)*	22.28 ± 8.79 *5.56, (3.16, 17.83)*	*0.091*
**IL 6 ***0 - 3.4 pg/ml *	172.53 ± 32.81 *77.5 (32.20, 160.75)*	2638.10 ± 1162 *533.0 (72.4, 2059)*	*0.001*
**CRP ***0 - 0.5 mg/dl *	13.74 ± 1.21 *12.85 (6.13, 18.68)*	13.94 ± 1.93 *13.65 (6.20, 18.15)*	*0.833*

ICU scores			

SAPSII	47.2 ± 10.6 *49.0 (32.0, 65.0)*	54.2 ± 12.3 (n.s.) *55.0 (43.8, 64.7)*	*0.167*
SOFA	10.6 ± 4.6 *11.0 (7.0, 14.0)*	14.6 ± 3.6 *14.5 (12.0, 16.8)*	*0.001*

Coagulation variables			

INR ***0.89 - 1.11***	1.31 ± 0.06 *1.19 (1.11, 1.40)*	1.64 ± 0.14 *1.49 (1.24, 1.94)*	*0.006*
Thrombin time	24.0 ± 2.0	23.4 ± 3.2	
***14 - 21 s***	*19.0 (15.0, 25.1)*	*17.4 (16.1, 27.5)*	0.955
Fibrinogen	498 ± 33	414 ± 63	
***210 - 400 mg/dl***	*512 (251, 676)*	*323 (231, 662)*	*0.159*
Platelets	154 ± 14	72 ± 11	
***160 - 350/nl***	*131 (72, 224)*	*65 (44, 95)*	*<0.001*

Shown are the impedance aggregometry findings obtained at the day of diagnosis with the activators adenosine diphosphate (ADP), arachidonic acid (AA), collagen (Col) and thrombin receptor activating peptide (TRAP). For comparison, the findings with the conventional sepsis markers procalcitonin (PCT), interleukin 6 (IL-6), and C-reactive protein (CRP) are given. Values are given as mean and standard error of the mean as well as median and quartiles, for statistical evaluation the Mann-Whitney-test was used.

**Table 5:** ROC-curve statistics for impedance aggregometry, sepsis markers, ICU-scores and coagulation variables for the prognosis of severe sepsis

Prognosis of sepsis Test	AUC	Confidence interval	Significance level
**Aggregometry**			

ADP	0.700	0.582 - 0.818	0.008
AA	0.626	0.488 - 0.764	0.093
Col	0.734	0.607 - 0.862	0.002
TRAP	0.756	0.641 - 0.871	0.001

**Sepsis Marker**			

PCT	0.617	0.254 - 0.511	0.117
IL6	0.731	0.126 - 0.413	0.002
CRP	0.504	0.353 - 0.639	0.956

**ICU scores**			

SAPSII	0.605	0.267 - 0.522	0.160
SOFA	0.732	0.154 - 0.382	0.002

**Coagulation variables**			

INR	0.719	0.569 - 0.868	0.006
Thrombin time	0.505	0.347 - 0.662	0.955
Fibrinogen	0.613	0.224 to 0.549	0.159
platelets	0.752	0.135 to 0.361	0.001

Area under curve, asymptotic confidence interval as well as asymptotic *P*-values are given for impedance aggregometry using the activators adenosine diphosphate (ADP), arachidonic acid (AA), collagen (Col) and thrombin receptor activating peptide (TRAP), as well as for the classical biomarkers procalcitonin (PCT), interleukin 6 (IL-6) and C-reactive protein (CRP), the SAPSII- and SOFA-score and coagulation variables.

**Figure 4:**
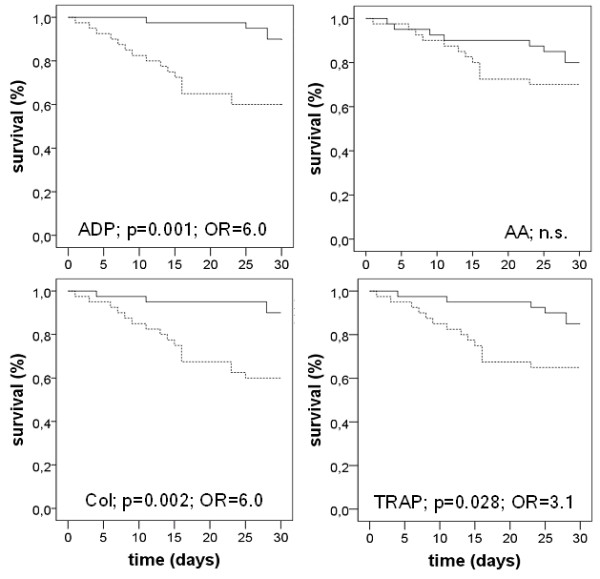
**Kaplan-Meier-analysis demonstrating 30-day survival of severe sepsis in patients in dependence on impedance aggregometry findings**. As the activators adenosine diphosphate (ADP), arachidonic acid (AA), collagen (COL) and thrombin receptor activating peptide (TRAP) were used. Moreover, the significance levels and the odds ratios (OR) are given.

To investigate the potential of impedance aggregometry as a predictor for the prognosis of severe sepsis, Kaplan-Meier analysis was performed. The results, shown in Figure 4, demonstrate that mortality differs in patients exhibiting lower and higher platelet function than the median, respectively. The best prediction of outcome was achieved by collagen-test and ADP-test with an odds-ratio of 6.0. Mortality was 40% in those patients with reduced platelet function, but only 10% in those patients exhibiting better aggregation. Impedance aggregometry using thrombin receptor activating peptide as the activator allowed differentiation between good and poor prognosis, although with a lower odds ratio (OR 3.1). Mortality differences were not detectable when arachidonic acid was used as the activator. Among the conventional biomarkers of sepsis, an interleukin 6 concentration showed significant differences between survivors and non-survivors, while procalcitonin and C-reactive protein failed.

Multivariate analyses were performed using logistic regression to evaluate which of the impedance aggregometry activators is an independent predictor of outcome. Procalcitonin, interleukin 6 and C-reactive protein as well as the results of impedance aggregometry obtained with one of the four activators were included in the analysis. The results of these analyses demonstrate that aggregometry findings obtained with ADP, collagen and TRAP were independent predictors of survival in severe sepsis (ADP: *P *= 0.049, collagen: *P *= 0.033; thrombin receptor activating peptide: *P *= 0.041). Impedance aggregometry using arachidonic acid as the activator failed as a predictor.

**Figure 5:**
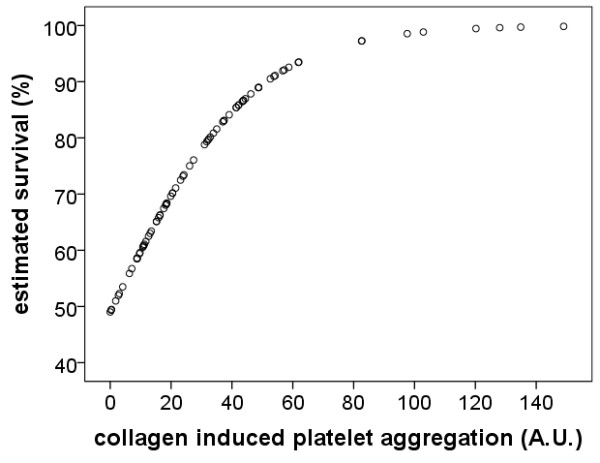
**Relation between platelet aggregation and estimated survival of patients with severe sepsis**. Values were calculated from the results of the logistic regression analysis obtained from collagen activated impedance aggregometry.

To determine the best predictor of survival in severe sepsis, logistic regression analysis was performed. Impedance aggregometry findings obtained with the four activators and the conventional biomarkers were included in one analysis. The results demonstrate that impedance aggregometry with collagen as the activator was the best and an independent predictor of survival in severe sepsis (*P *= 0.03). In Figure 5, the estimated survival as a function of the collagen induced platelet aggregation findings derived from logistic regression analysis is shown.

### Comparison of impedance aggregometry findings with platelet count

Impedance aggregometry findings might be affected by the platelet count. To exclude that impedance aggregometry simply reflects platelet count, we compared both variables. Platelet count was 196 (154, 245)/nl in postoperative patients and 107 (57, 187)/nl in patients with severe sepsis (medians and quartiles, Table 2). Comparison of impedance aggregometry findings with platelet count demonstrated only low correlations with the four activators in postoperative patients (r^2^: range 0.297 to 0.508) and patients with severe sepsis (r^2^: range 0.287 to 0.441). Correlations among the four impedance aggregometry activators were between 0.320 and 0.671 (r^2^) in postoperative patients and between 0.253 and 0.566 (r^2^) in patients with severe sepsis. In order to determine whether impedance aggregometry or platelet count is the better predictor of severe sepsis in critical illness, multivariate analysis was performed. In the logistic regression analysis, impedance aggregometry using four activators and platelet count was included. The results of the analysis demonstrate that platelet aggregometry findings using collagen and thrombin receptor activating peptide as the activators are the best and independent predictors of severe sepsis in critical illness (*P *= 0.009 and *P *= 0.002, respectively).

In non-survivors, platelet count was 65 (44, 95)/nl at the first day of sepsis, while 131 (44, 95)/nl platelets were determined in the survivors (medians, quartiles, *P *= 0.001). Multivariate analysis was used to determine whether platelet function or platelet count is the better predictor of outcome. Logistic regression analysis, including platelet count and platelet aggregation findings obtained with the four activators, demonstrated that platelet aggregation induced by collagen is the best and an independent predictor for survival in severe sepsis (*P *<0.001).

### Conventional coagulation variables and scores as biomarkers for the diagnosis and prognosis of severe sepsis

Among the coagulation markers INR, thrombin time and fibrinogen, only INR showed moderate predictive value for both diagnosis and prognosis of severe sepsis in the present study. Thrombin time and fibrinogen did not differ significantly (Tables 2, 3, 4, 5). The SAPS II and SOFA score were both excellent markers for the diagnosis of severe sepsis, but only the SAPS II score was capable of differentiating between survivors and non-survivors of severe sepsis (Tables 2, 3, 4, 5).

## Discussion

The results of the present study demonstrate that impedance aggregometry findings are good predictors of the diagnosis of severe sepsis in critical illness and the outcome of severe sepsis. Notably, impedance aggregometry findings using collagen as the activator proved to be a better biomarker for both diagnosis of severe sepsis in critical illness and survival of severe sepsis than procalcitonin, interleukin 6, C-reactive protein and platelet count.

Impedance aggregometry using the Multiplate^® ^device has been demonstrated to be predictive of bleeding in cardiac surgery as well as thrombosis and early mortality in percutaneous coronary and neuroradiology stenting, respectively [16,17]. Unlike the Born aggregometry, the method relies on measurement of impedance changes caused by platelet aggregation on electrodes in whole blood samples [15,22]. Thus, no centrifugation step is necessary and measurement can be done in the intensive care setting. Pipetting with an automated pipette is guided by a computer menu. Within 12 minutes, results are obtained and hands-on time is about 6 minutes. Dual measurements in each vial allow automatic quality control. Costs per measurement are actually below 10€. The measurement of platelet function in whole blood samples might be advantageous: the presence of the patient´s plasma, including all mediators and all circulating blood cells, in a certain pathophysiological setting might affect the results, while the Born aggregometry is often performed in a standardized citrated medium and, thus, is more artificial. Moreover, the Born aggregometry requires a specialized laboratory.

The finding that platelet aggregation is a valuable biomarker for both diagnosis and prognosis of severe sepsis is in close agreement with the changes observed in the hemostatic system in severe sepsis. It has convincingly been demonstrated that the DIC score is a predictor of outcome [23]. Moreover, we recently demonstrated that inhibition of clot lysis, as determined by thromboelastometry, is a good marker for severe sepsis in critical illness [24]. Additionally, we demonstrated that a hypocoagulative status, determined by the same method, is a good predictor of survival in severe sepsis [25]. Although the present study was not designed to define mechanisms of platelet hyporesponsiveness, some suggestions can be made. Disseminated intravascular coagulation in severe sepsis is caused by the expression of tissue factor on the surface of monocytes and the procoagulatory shift of the endothelium [2]. As a consequence, coagulation occurs in microcirculation and leads to the deposition of fibrin and platelets. Accordingly, the concentration of coagulation factors will decrease and the population of well functioning platelets will be sequestered in the capillaries [26]. Thus, the samples drawn from patients with severe sepsis might show platelet aggregation of the remaining hyporesponsive platelets. Moreover, the common activators of platelet aggregation, which are ADP, thrombin and collagen have all been demonstrated to be altered in sepsis [27-30]. Homologous and heterologous desensitization of signal transduction pathways might, therefore, serve as an explanation for altered platelet reactivity in severe sepsis [31].

For the prediction of diagnosis, as well as outcome of severe sepsis collagen, ADP and thrombin receptor activating peptide can be used as the activators for impedance aggregometry. In contrast, arachidonic acid was only a moderate biomarker for the diagnosis of severe sepsis in critical illness and failed to predict outcome. The reasons for the discrepancy observed between the diagnostic and prognostic value of the activators used are not known. However, recent evidence deriving from neutrophils, showing multiple alterations in signal transduction during sepsis, favors the point of view that changes in signal transduction might be responsible for the observed changes in platelet aggregation [32]. Interestingly, the median values of the postoperative patients were near the lower normal range given by the manufacturer of the Multiplate^® ^device; so it can be hypothesized that surgery *per se *might slightly reduce platelet function. However, the present study was not designed to answer this question.

In contrast to Born aggregometry, the platelet count of samples for impedance aggregometry is not adjusted to a certain value [22]. Instead, whole blood samples are subjected to impedance aggregometry without normalization of platelet count. Recently, impedance aggregometry has been demonstrated to be affected by severe thrombocytopenia. However, correlation of platelet count with impedance aggregometry findings was low and it was suggested that the method reflects over-all aggregability of platelets [33]. Our results are in agreement with this study: the correlation between platelet count and impedance aggregometry was low and the striking differences in the predictive value of the activators suggest that the method indeed determines the aggregability of a sample and not simply the platelet count. Moreover, this point of view is supported by the results of the multivariate analyses showing that platelet aggregation findings using collagen as the activator is the best and an independent predictor of outcome.

Platelets have functions in sepsis which are beyond hemostasis and are related to immunologic functions. Platelets have been shown to have phagocytic-like functions and can engulf bacteria, viruses and foreign bodies and may participate in immune defense [7]. Moreover, it has been suggested that bacteria can hook on and ride on platelets to a vessel injury when the platelet surface is modified by bacterial neuraminidase [3,34]. To counteract this harmful mechanism, the Ashwell-Morell receptor has evolved, which eliminates these modified platelets in the liver [35]. In addition, platelets have been demonstrated to glom to neutrophils, when activated by blood stream infection via TLRs. As a result, neutrophils release webs of DNA, so-called neutrophil extracellular traps [36]. Taken together, the above mechanisms demonstrate the tight involvement of platelets in the defense of pathogens.

In recent attempts, platelet function inhibition in experimental sepsis has been demonstrated to exert beneficial effects [37]. Moreover, acetylsalicylic acid treated patients have recently been shown to have a better prognosis in sepsis in an observational study [38]. The reduced aggregability of patients with severe sepsis, especially with poor prognosis, suggests that platelet inhibition might be performed at a calculated risk. In this regard, impedance aggregometry might serve not only as a monitoring tool for the estimation of sepsis severity, but might guide an eventual antiplatelet and eventual immune modulatory therapy.

The present study has limitations. Although platelet function was obtained in 130 patients, the sample size of 80 patients with severe sepsis, 20 of whom died, was limited. Moreover, specifity of the impedance aggregometry as a biomarker has to be verified with different control groups. Furthermore, it is important to state that the cut-off value used for Kaplan-Meier analysis and determination of odds ratios in the present study is identical to the median of the sepsis patients in the same cohort. It is thus necessary to determine the validity of the cut-off in further studies. Thus, the present study might present a first framework for further studies on impedance aggregometry in patients with severe sepsis.

## Conclusions

The results of the present study demonstrate that platelet aggregation in whole blood samples, determined on the first day of admission on the intensive care unit, might serve as a new and reliable biomarker for the diagnosis and the outcome of severe sepsis. The early changes in platelet aggregation at the first day of severe sepsis suggest an important pathophysiological role of platelets in severe sepsis.

## Key messages

• Platelet aggregation, as determined by impedance aggregometry at Day 1, is markedly lower in patients with severe sepsis than in postoperative patients.

• Platelet aggregation in non-survivors of severe sepsis is lower than in survivors.

• Impedance aggregometry is a better biomarker for the diagnosis and prognosis of severe sepsis than conventional biomarkers and platelet count.

## Abbreviations

ADP: adenosine diphosphate; A.U.: arbitrary units; CRP: C-reactive peptide; IL-6: interleukin 6; INR: international normalized ratio; ROC-curve: receiver operating characteristic curve; SAPS II: simplified acute physiology score; SOFA: sequential organ failure assessment score; TLR: Toll-like receptor; TRAP: thrombin receptor activating peptide 6

## Competing interests

KG received payment for lectures, including service on speakers' bureaus from Verum Diagnostica GmbH, Munich, Germany, Instrumentation Laboratory, Kircheim, Germany, and Triolab, Copenhagen, Denmark. The other authors declare that they have no competing interests.

## Authors' contributions

Conception of the study was done by MH. Data acquisition of patients was performed by MA. MH and KG performed impedance aggregometry analyses. Analysis of data and drafting of the manuscript was done by MH. All authors critically revised and approved the manuscript.
